# Standardized analysis of syndesmosis stability in ankle trauma with an innovative syndesmosis-test-tool: a biomechanical study

**DOI:** 10.1038/s41598-024-51872-4

**Published:** 2024-01-18

**Authors:** Felix Christian Kohler, Jakob Hallbauer, Lea Herrmann, Bernhard Wilhelm Ullrich, Uta Biedermann, Britt Wildemann, Gunther Olaf Hofmann, Roland Ramm, Mark Lenz, Ivan Marintschev, Philipp Schenk

**Affiliations:** 1 Department of Trauma, Hand and Reconstructive Surgery, Jena University Hospital, Friedrich Schiller University Jena, 07747 Jena, Germany; 2 Department of Trauma and Reconstructive Surgery, BG Klinikum Bergmannstrost Halle gGmbH, 06112 Halle, Germany; 3 Institute of Anatomy I, Jena University Hospital, Friedrich Schiller University Jena, 07743 Jena, Germany; 4 Fraunhofer Institute for Applied Optics and Precision Engineering (IOF), Albert-Einstein-Str. 7, 07745 Jena, Germany; 5 Department of Science, Research and Education, BG Klinikum Bergmannstrost Halle gGmbH, 06112 Halle, Germany

**Keywords:** Trauma, Fracture repair

## Abstract

When treating ankle fractures, the question of syndesmosis complex involvement often arises. So far, there is no standardized method to reliably detect syndesmosis injuries in the surgical treatment of ankle fractures. For this reason, an intraoperative syndesmosis-test-tool (STT) was developed and compared to the recommended and established hook-test (HT). Tests were performed on cadaveric lower legs (n = 20) and the diastasis was visualized by 3D camera. Tests were performed at 50, 80, and 100 N in native conditions and four instability levels. Instability was induced from anterior to posterior and the reverse on the opposite side. The impact on diastasis regarding the direction, the force level, the instability level, and the device used was checked using a general linear model for repeated measurement. The direction of the induced instability showed no influence on the diastasis during the stability tests. The diastasis measured with the STT increased from 0.5 to 3.0 mm depending on the instability, while the range was lower with the HT (1.1 to 2.3 mm). The results showed that the differentiation between the instability levels was statistically significantly better for the developed STT. The last level of maximum instability was significantly better differentiable with the STT compared to the HT. An average visualizable diastasis of more than 2 mm could only be achieved at maximum instability. In conclusion, the newly developed STT was superior to the commonly used HT to detect instability.

## Introduction

During the stabilization of ankle fractures, the question usually arises as to whether syndesmosis stability is sufficient or whether additional stabilization is necessary^1^. To prevent long-term posttraumatic complications, relevant instability should not be missed and be adequately addressed, for example by suture and bracement of the syndesmosis, syndesmotic screws, or suture button stabilization^2^. However, there is a discussion about whether an injury to the anterior inferior tibiofibular ligament (AITFL) alone requires syndesmosis stabilization^3^. To date, the question has not been conclusively answered as to when a syndesmosis injury becomes relevant to justify additive stabilization. Without doubt, stabilization should be performed if there is the slightest doubt about the stability of the syndesmotic complex^4^.

To identify syndesmosis injury-related instability clinical signs of examination as well as imaging such as MRI, stress images, or ultrasound are used^5^. The measurement of syndesmotic parameters such as medial or tibiofibular clear space under stress recordings of the ankle is of limited value because of its dependence on ankle rotation^4,6^. To avoid differences in the measured distances due to rotation, repeated exact adjustment of the mortise view would be necessary for all cases, but often cannot be achieved practically^6^. The sensitivity and specificity of radiographs can be further enhanced by stress imaging and provide important clues, but they often cannot provide a definitive diagnosis^7–9^. An additional CT scan has a major role in surgical planning and can provide indirect evidence^4,10,11^. However, numerous anatomic variants of the tibiofibular distance also make the diagnosis uncertain and necessitate comparison to the healthy opposite side in all cases^7,12^. MRI shows nearly perfect specificity and sensitivity close to 100% for syndesmotic injuries^7^. However, MRI is expensive and not ubiquitously and rapidly available preoperatively^13^. Both CT and MRI are static examinations that do not allow dynamic investigation of possible instability^4^. Intraoperative arthroscopy has a sensitivity and specificity of 100%, but is invasive, not ubiquitously available, and requires experience and appropriate skills^7^.

Intraoperative lateral stress tests such as the Hook-Test (HT) or external rotation stress test (ERST) under X-ray inspection are common to detect instabilities^14,15^. The morphology of the injury can also provide clues to the extent of damage to the syndesmotic complex. According to Lauge-Hansen, complete disruption of the syndesmotic ligaments is always expected in stadium 4 trimalleolar injuries for pronation or supination external rotation traumas^4,16^. If there is any doubt about the decision to perform additional stabilization, the anterior syndesmosis can be visualized by lateral approach to the distal fibula or arthroscopically^17^.

However, for intraoperative testing, some serious issues exist regarding the reliability and interpretation of the applications^18^. In a recent study, we showed that intraoperative HT has poor interrater reliability and fails to achieve a relevant increase in diastasis^18^. It has also been shown that the syndesmotic complex has a high degree of individual laxity and thus a highly interindividual native diastasis of the ankle mortise^18^. Also, the external rotation stress test showed poor, sometimes worse results than the HT^4,19,20^. The major problem with the stress tests is the large differences in the applied forces^18,21^. A force between 50–100 N is recommended^4,18^. Furthermore, Candal-Couto et al. found that fibular motion in the sagittal plane was consistently greater than motion in the coronary plane, so distal tibiofibular instability may be more appropriate to assess in the sagittal plane using the HT^4,22^.

Currently, there is no standardized recommendation for the force or direction in which the HT should be performed^18,21^. This makes it clear that it is extremely difficult to compare results between different studies regarding syndesmotic instabilities.

For this purpose, an innovative syndesmosis-test-tool (STT) was developed and compared to the established HT, which enables standardized intraoperative applied force (50–100 N) to test the stability of ankle injuries. Both tests were examined by comparing the diastasis for native to maximum ankle instability in a biomechanical cadaver study.

## Materials and methods

### Cadaveric specimens

Human lower legs (10 pairs of fresh-frozen intact lower legs: 7 males, 3 females, mean age 87 years; range 74–94 years) from voluntary body donors from the Institute of Anatomy I at the university were used. No previous accidents or operations were known. The samples were thawed for 18 h at room temperature. The soft tissue in the malleolar region was removed, protecting the ligamentous parts. This included the ligamentous structures of the syndesmosis as well as the deltoid ligament in the region of the medial malleolus and the lateral ligamentous structures in the region of the lateral malleolus.

The study was approved by the local ethics committee (approval number: 2021–2431 material) and was conducted in line with the principles of the Declaration of Helsinki.

### Description of the developed STT

**Figure 1:**
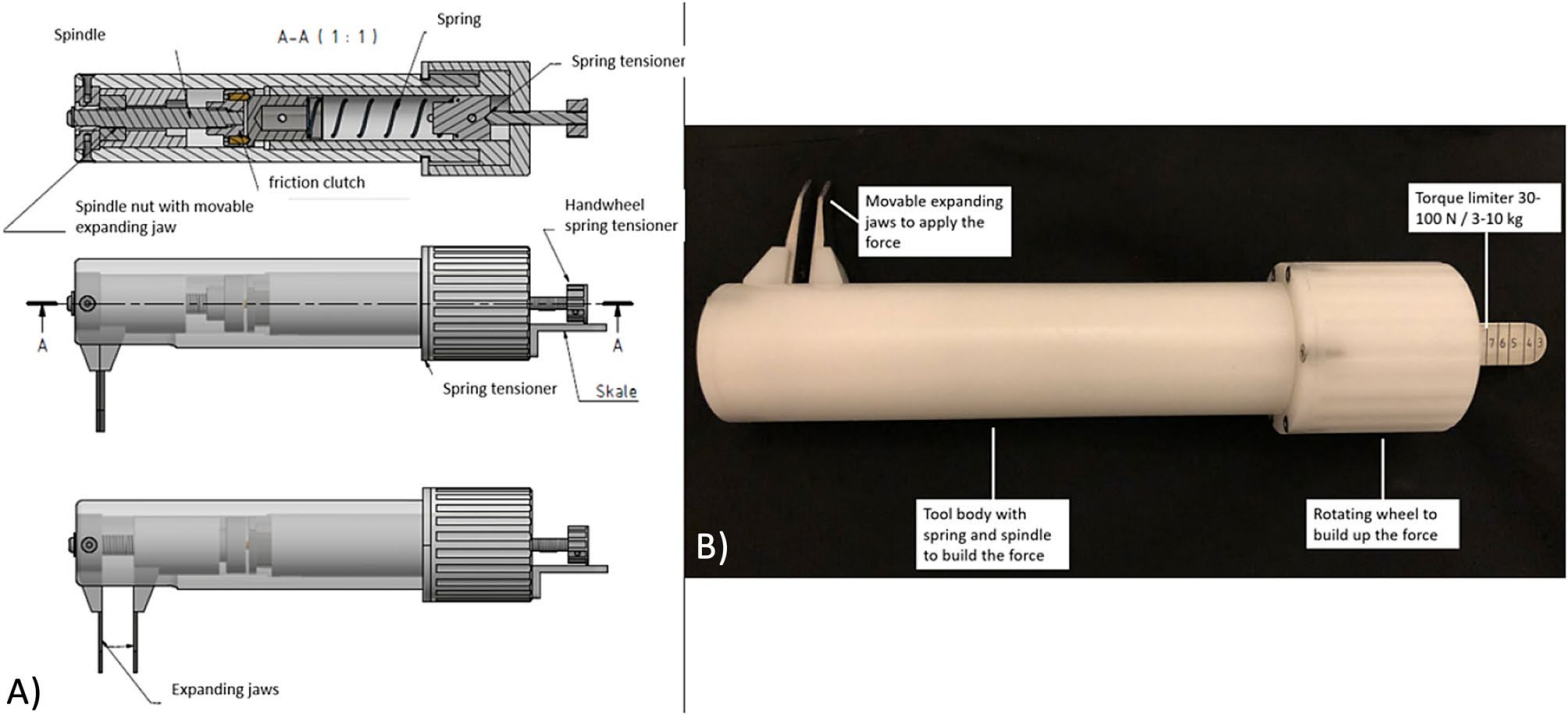
The figure shows the syndesmosis-test-tool with the most important components. (**A**) illustrates the construction plan of the syndesmosis-test-tool and how the force can be built up with the help of a spring inside the body. (**B**) shows the syndesmosis-test-tool as it was used in the study. The accuracy of the force development was checked in a test series.

**Figure 2:**
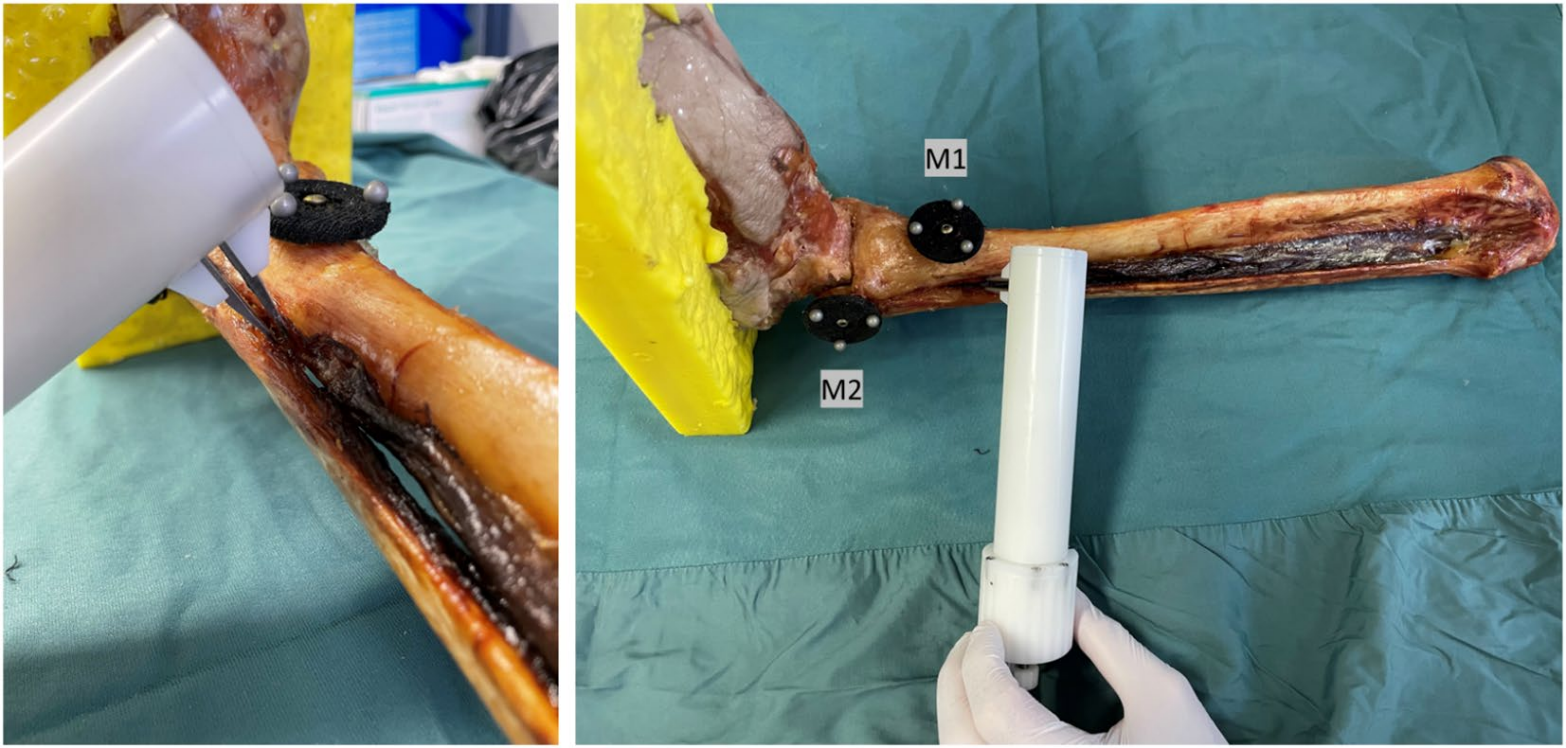
The use of the syndesmosis-test-tool at the syndesmotic level is shown. In contrast to the Hook-Test, where a hook is used to pull at a 90° angle to the lower leg, the force is applied at an angle of approximately 45° with the syndesmosis-test-tool. The marker plates consisted of three reflective spheres each and were labeled M1 (tibia) and M2 (fibula).

To be able to apply a standardized force and thus stress the syndesmosis, a tool was developed in cooperation with the research workshop of the university (Fig. 1). This consists of two metal plates that can be inserted in the space between the tibia and fibula. A spindle is built into the body of the tool, which in turn can be used to build up a defined force via a rotary wheel at the other end. A torque limiter can limit the maximum force to be applied via another wheel mounted at the end of the rotary wheel. Forces between 50 and 100 N can be set here. After the set force is reached, no further force is built up via the rotary wheel. The tool can thus be used to apply a force to be set between the fibula and the tibia. The metal plates and thus also the force vector are oriented slightly obliquely and thus following the orientation of the two bones to each other (about 45°), with the fibula lying dorsally to the tibia (Fig. 2).

### Description of the intraoperative HT

A commercially available portable digital scale was attached to the fibula 2 cm proximal to the anterior syndesmosis. It was pulled horizontally (90 degrees to the fibula shaft axis) laterally on the fibula while the applied force was constantly monitored.

### Biomechanical evaluation of the syndesmotic diastasis

To record the induced diastasis between fibula and tibia by the forces applied in both tests, a 3D camera system of the Fraunhofer IOF was used (optical 3D measurement system »kolibri CORDLESS« with two stereo cameras of resolution 2048 × 1280 pixels, measurement uncertainty 20–100 µm, 30 frames per second for marker tracking). Two passive optical marker plates were applied to the distal fibula and tibia at the level of the syndesmosis. The marker plates consisted of three reflective spheres each and were labeled M1 (tibia) and M2 (fibula) (Fig. 2). The test specimens were fixed proximally to the tibial plateau and distally to the foot to perform the load tests. Initially, the load tests were performed with the two tools on the intact lower legs without destroying the syndesmosis. Forces of 50, 80, and 100 N were applied at each test step. The STT was inserted 2 cm proximal to the level of the anterior syndesmosis between the fibula and tibia and the rotary wheel was operated until the torque sensor was triggered and the previously set force was reached. The change in diastasis or the movement of the two markers (M1 and M2) was monitored with the 3D camera during this process. The tests were first started by being randomized with the STT or the HT and after were performed consecutively.

Each pair's left and right legs were randomly assigned to either of two groups (anterior to posterior or posterior to anterior). In the first step (1.), all specimens were tested natively without injury to document the native diastasis of the syndesmosis as the initial condition. Steps 2–5 follow as destabilization steps.

In the first group (anterior to posterior), the syndesmosis was destabilized in stages according to the following protocol (start with 2. step after initial native testing):

2. Cutting the anterior syndesmosis ligament (AITFL).
3. Cutting the intermediate syndesmotic ligament
4. Osteotomy of the posterior tibial rim (bony fixation of the PIFTL)
5. Cutting the deltoid ligament.

The ligamentous structures were cut with a standard surgical scalpel. An oscillating saw was used to osteotomize the posterior malleolus.

In the second group (posterior to anterior), the protocol was performed in reverse order as follows (start with 2. step after initial native testing):

2. Cutting the deltoid ligament.
3. Osteotomy of the posterior tibial rim (bony fixation of the PIFTL)
4. Cutting the intermediary syndesmotic ligament
5. Cutting the anterior syndesmotic ligament (AITFL)

Following each of the four cutting steps, the STT and HT were used to apply a force of 50, 80, and 100 N respectively. The diastasis was continuously monitored with a 3D camera. A relevant threshold value of the relative movement between the two bones of > 2 mm was determined. This value is based on a native physiological diastasis of approximately 1 mm in the mediolateral direction, as reported in the literature^23,24^.

The databases regarding the HT data, used in this work, have already been examined and published concerning reliability^18^.

### Statistical methods

In the first step, a general linear model (GLM) for repeated measures was used to detect the impact of the direction of stepwise-induced instability. Therefore, as a between-subject factor, the variable with the coded information of the direction of the stepwise performed instability (from anterior to posterior or from posterior to anterior) was used. The maximum diastasis, measured repeatedly by the STT and the HT (device) during the applied force (3 levels), and the instability (5 levels), were used as within-subject factors (repeated measures). If the direction of stepwise induced instability showed no significance as a main effect and in the interaction effect, a second GLM was used, in which the direction was omitted to increase the power of the analysis.

The Greenhouse–Geisser *p* value is used for the main and interaction effects (force and instability) in cases in which the Mauchly test for sphericity shows significance. For the main effects (force, instability, and device), the effect sizes (ES) are given in partial Eta squares (p.Eta^2^) in addition to the *p* value. Values of 0.01, 0.06, or 0.14 suggest small, medium or large effects, accordingly^25^. Due to multiple tests and the resulting accumulation of alpha errors, Bonferroni-corrected pairwise post-hoc comparisons were performed. The pairwise post-hoc comparisons between the instability levels, separately for each device, are supplemented by Cohen's d^26^ effect sizes in addition to the *p* values for better differentiation and interpretation: d = 0.2 small effects, d = 0.5 medium effects, and d = 0.8 large effects.

The results are shown as mean values and 95% confidence intervals as error bars for visual comparison. This means that samples with non-overlapping error bars are significantly different, with *p* ≤ 0.05.

The significance level was set at *p* ≤ 0.05. SPSS version 27 software (IBM Corp. Released 2020. IBM SPSS Statistics for Windows, version 27.0. Armonk, NY: IBM Corp) was used for the statistical analyses. The statistics were performed by one of the authors (P.S.), a biostatistician at the Department of Science, Research and Education.

### Informed consent

Informed consent was obtained from all subjects involved in the study. The data presented in the study are stored on secure servers at University Hospital Jena. Donor consent forms are stored at the Anatomical Institute of the University Hospital in Jena. The data are available on request from the corresponding author.

## Results

### General information

The stability of the syndesmosis was tested with ten human cadaveric lower leg pairs (n = 20) with increasing force (50, 80, and 100 N) at each level of instability (from intact to maximum instability). The instability of the syndesmosis was generated from two different directions. The stabilizing ligaments were serially cut in either a posterior-to-anterior or anterior-to-posterior order.

### Validity of the syndesmosis-test-tool

The validity of the STT for 50 N, 80 N, and 100 N was checked in a series of 10 measurements each, resulting in 51 ± 1.4 N, 80 ± 1.7 N, and 101 ± 2.0 N.

### Direction of destabilization

The direction of the generated instability by cutting the ankle ligaments stepwise from anterior to posterior or from posterior to anterior showed no significant impact on the diastasis (*p* = 0.853, Table 1). In Table 1, the *p* values and effect sizes as p.Eta^2^ of the general linear model (GLM) are given. Because the direction of the induced instability had no significant impact on the diastasis, it was not taken into account in further analyses to increase the statistical power. The results of the GLM are given in Table 2 as *p* values, and the effect sizes for the main effects and interaction without consideration of the direction of the instability are listed.

**Table 1:** Statistical results of the GLM data to detect the impact of the direction (a-p or p-a) of induced instability on the diastasis.

	*p* value	p.Eta^2^
Direction (a-p vs. p-a)	0.853	0.002
Device * Direction	0.280^#^	0.058
Force * Direction	0.473^#^	0.032
Instability * Direction	0.676^#^	0.018
Device * Force * Direction	0.681^#^	0.013
Device * Instability * Direction	0.811^#^	0.006
Force * Instability * Direction	0.139^#^	0.085
Device * Force * Instability * Direction	0.570^#^	0.035

^#^Adjusted *p* values by Greenhouse–Geisser. Direction: a: anterior, p: posterior.

**Table 2:** Statistical results of the GLM without consideration of the direction of induced instability.

	*p* value	p.Eta^2^
Device	**0.047**	0.175
Force	** < 0.001**^#^	0.392
Instability	** < 0.001**^#^	0.750
Device * Force	**0.002**^#^	0.310
Device * Instability	**0.003**^#^	0.299
Force * Instability	0.721^#^	0.023
Device * Force * Instability	**0.025**^#^	0.123

^#^Adjusted *p* values by Greenhouse–Geisser.

Significant values are in bold.

### Measurements of the diastasis generated by the syndesmosis-test-tool and hook-test

**Figure 3:**
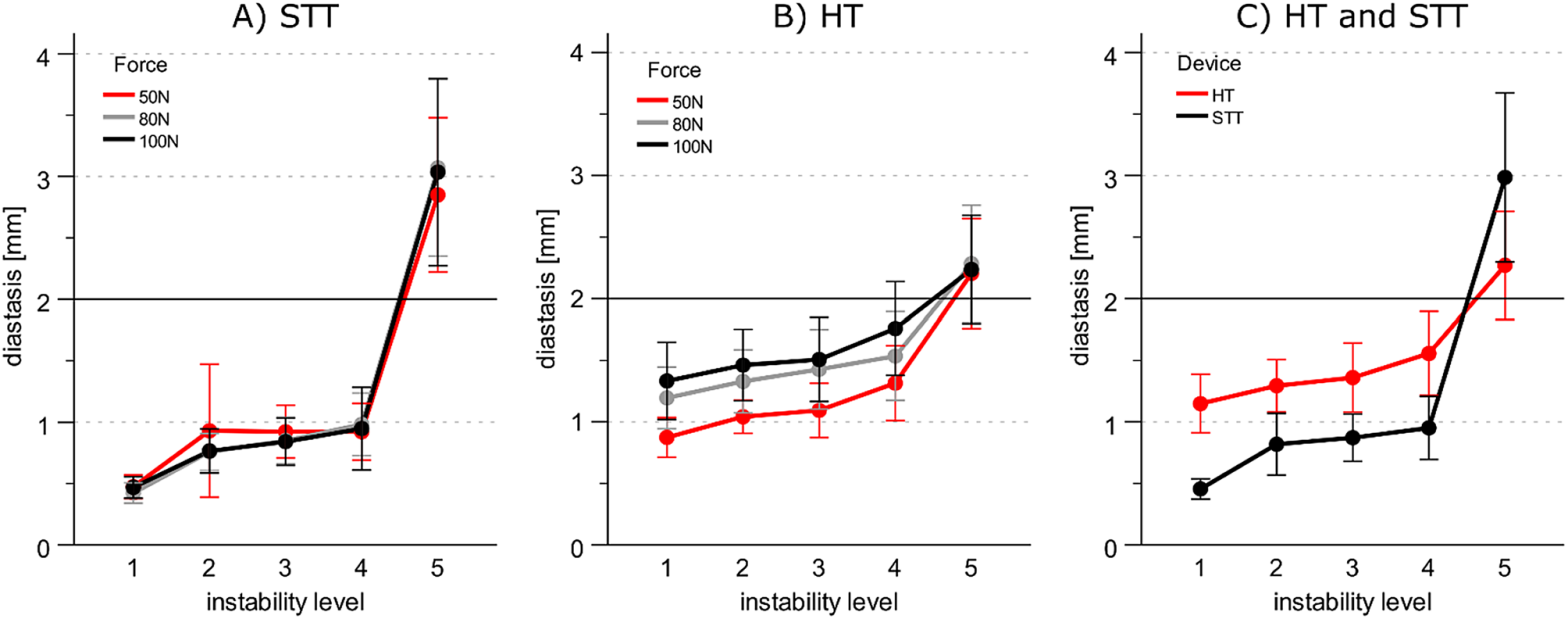
Level of instability and applied forces (in color) with the corresponding diastasis in (**A**) for the syndesmosis-test-tool (STT) and in (**B**) for the Hook-Test (HT). In (**C**), the comparison of the diastasis for the syndesmosis-test-tool (STT) and the hook-test (HT) regardless of the force, is shown. Solid horizontal line: instability threshold of 2 mm. The diastases are given as mean ± 0.95 confidence interval as error bars (n = 20). Samples with non-overlapping error bars differ significantly, with *p* ≤ 0.05.

The HT showed an overall larger mean diastasis with 1.5 ± 0.8 mm, compared to the STT with 1.2 ± 1.2 mm (*p* = 0.047), regardless of the applied force, the instability level, or the direction of the induced instability. The diastasis measured with the STT ranged from 0.5 ± 0.2 mm to 3.0 ± 1.6 mm depending on the instability level, while the range with the HT was 1.1 ± 0.4 mm to 2.3 ± 1.0 mm (Fig. 3, Table 3).

**Table 3:** Values of diastasis for both devices, syndesmosis-test-tool (STT) and hook-test (HT) for each level of instability. The diastasis generated is given in mm as the mean ± standard deviation (n = 20).

Level of instability	STT	HT	P
1	0.5 ± 0.2	1.1 ± 0.6	** < 0.001**
2	0.8 ± 0.8	1.3 ± 0.6	**0.003**
3	0.9 ± 0.4	1.4 ± 0.7	**0.003**
4	1.0 ± 0.6	1.6 ± 0.8	**0.002**
5	3.0 ± 1.6	2.3 ± 1.0	0.101

Significant values are in bold.

The diastasis measured with the HT (mean values of all instability levels: 50 N 1.3 ± 0.8 mm, 80 N 1.6 ± 0.9 mm, 100 N 1.7 ± 0.9 mm) depended on the applied forces (50 N vs. 80 N *p* < 0.001, 50 N vs. 100 N *p* < 0.001, 80 N vs 100 N *p* = 0.040), regardless of the instability level (Fig. 3A). In contrast, the STT showed no force-dependent diastases with *p* = 1.000 for each post hoc pairwise comparison (Fig. 3B).

By comparing both devices for each instability level, the HT showed significantly greater diastasis in each instability level (level 1 *p* < 0.001, level 2 *p* = 0.003, level 3 *p* = 0.003, level 4 *p* = 0.002, Table 3) compared to the STT, except at the maximal instability (instability level 5, *p* = 0.101, Table 3). The pairwise comparisons of the diastases between both devices for each instability level are given in Table 3.

Both devices showed different slopes depending on the instability level, which is statistically given as an interaction between the device and instability level with *p* = 0.003 in Table 2. This means, that HT and STT produced different diastases depending on the instability level. However, statistical analysis revealed a very comparable frequency of reaching significance for both devices, except that the STT showed significance between native condition (level 1) and instability level 3 (*p* < 0.001), in contrast to the HT (*p* = 0.140, Table 4). With a moderate effect (Cohen's d greater 0.5) that distinguishes between different instability levels, the effect sizes for STT are larger compared to HT (Table 4). The greater the effect size, the greater the difference in diastasis between the two instability levels.

**Table 4:** Results of post hoc pairwise comparisons between the instability levels for each device, as *p* values and the effect sizes (Cohens’d with d = 0.2 show small effect, d = 0.5 moderate effect and d = 0.8 large effect, n = 20).

Level of instability		STT		HT	
		p	Cohens’d	p	Cohens’d
**1**					
	2	0.076	0.62	0.076	0.25
	3	** < 0.001**	1.04	0.140	0.32
	4	**0.002**	0.96	**0.031**	0.56
	5	** < 0.001**	1.50	** < 0.001**	1.11
**2**					
	3	1.000	0.09	1.000	0.11
	4	1.000	0.19	0.241	0.37
	5	** < .001**	1.33	** < 0.001**	1.02
**3**					
	4	1.000	0.15	0.903	0.26
	5	** < 0.001**	1.36	** < 0.001**	0.93
**4**					
	5	** < 0.001**	1.30	**0.001**	0.73

Significant values are in bold.

For visual comparison, Fig. 3C displays the diastasis of both devices in dependency on the instability level. The largest increase in diastasis between adjacent instability levels produced the STT from instability levels 4 to 5 with 2.0 ± 1.7 mm (*p* < 0.001). For the HT, the increase from instability level 4 to 5 of 0.7 ± 0.7 mm was also the greatest between adjacent instability levels within the HT (*p* = 0.001) but was almost three times smaller compared to the STT. The difference between native condition (level 1) and maximal instability (level 5) was 2.5 ± 1.5 mm for the STT and 1.1 ± 0.8 mm for the HT.

The effect sizes showed better discrimination between instability levels for the STT, than for the HT.

Visually discriminating in diastasis between the different instability levels is quite hard, despite its significance.

## Discussion

Due to the heterogeneous performance of the HT, regarding force applied by the surgeon and certain instability^18^ the aim was to compare a developed device that will lead to improved testing of ankle instabilities. In this study, a newly developed STT was compared with a widely used and recommended intraoperative test, the HT^27^. For this purpose, the generated diastasis was measured in ankle joints with different instabilities (from native stability to maximum instability in terms of Lauge-Hansen) by applying different force levels.

Summarizing our results, instability showed the largest impact on diastasis. Using the HT, applied force (whether it was pulled with 50, 80, or 100 N) matters, but not for the STT. Since we know that surgeons perform the HT with very different forces^18,21^, the STT provides a clear advantage here with its reliable application between 50–100 N compared to a non-standardized intraoperative HT.

The direction of the generated instability, increasing instability from posterior to anterior versus anterior to posterior, did not affect the generated diastases. Statistically, the STT showed a better differentiation between the instability levels, compared to the HT. In addition, the increase in diastases at maximum instability was three times greater with the STT than with the HT, and therefore the change in diastasis can be easily identified macroscopically.

The significant interaction between the applied force and the level of instability for the HT shows the dilemma of the HT. The induced diastasis depended on the force pulled by the HT and the instability level. This led to higher heterogeneity in detecting instability. The STT did not show force-dependent diastasis. Thus, the HT seems less reliable to detect instability, compared to the STT.

Failing the diagnosis of syndesmosis instability can have consequences such as early degenerative alterations of the upper ankle^7,28^. Patients with unstable syndesmosis injuries benefit from surgical stabilization in the long term^29^. On the other hand, stable injuries, for example, isolated to the AITFL, can be treated conservatively, and therefore not every injury to the syndesmosis needs to be treated surgically^30,31^. Preoperatively, radiographic measures on native radiographic imaging may indicate syndesmosis instability^7^. The determination of instability, especially when maximum instability is present, is mandatory to initiate adequate therapy. Due to the larger effect sizes and the more frequent significance (instability level 1 versus 3), the STT seems more suited to detect even partial injuries, compared to the established HT.

However, in absolute terms, except for level 5, the differences in diastases for instability levels 1 to 4 were less than 2 mm. These differences can hardly be detected visually under intraoperative X-rays. Unacceptable instability is usually assumed when the diastasis increases by at least 2 mm in the tibiofibular clear space^2,24^. The HT showed force-dependent diastasis and reached the commonly used sign of instability of 2 mm diastasis just in a few cases, even for the maximum instability. Nevertheless, it is possible to detect maximum instability with the HT. However, this requires pulling with appropriate force, which can be different for surgeons^18^. At maximum instability, the diastasis between the three forces did not differ, both for the STT and the HT. Here, the force does not seem to matter at this point. Even low forces of 50N are sufficient to detect the maximal instability for both devices.

For intraoperative testing, the literature gives advantages to the lateral stress test (= HT)^19,20^ others show that the ERST could be superior in the detection of instabilities^32,33^. These tests detect only high-grade injuries of all syndesmosis ligaments (AITFL, PITFL, IOL, possibly deltoid ligament) in the mortise view^18,32^. The performance of the tests with the individually very different applied forces is subject to large interindividual variations, as is the correct radiographic setting of the mortise view^18,21^.

An important difference between the two test methods was the application of force to the syndesmosis. With the HT, the force is usually applied at 90° to the shaft axis of the fibula^27^. However, whether the direction of the force applied laterally in the frontal plane is generally the appropriate direction for the HT is controversial^22^. With the STT, the force is built up by a spreading mechanism above the syndesmosis plane between the two bones. The oblique insertion of the STT (approx. 45 degrees compared to the HT, Fig. 3) between the fibula and tibia is due to both the anatomy and the design of the STT. The fibula lies dorsal to the tibia, and the STT is therefore inserted at an oblique angle. Due to this methodical difference, not only the coronal but also the sagittal translation is considered for the STT. This may explain the advantage shown in comparison to the HT. Further studies should investigate the direction of the force vectors for the intraoperative test methods on the diastasis produced.

Due to the use of cadaver specimens, our results cannot be transferred one-to-one to the intraoperative situation of patients with muscles, ligaments, and soft tissue tension. Nevertheless, it is the closest simulation possible in the laboratory. Tests with newly developed tools must first prove their validity in biomechanical cadaveric studies before they can be tested in vivo. The availability of body donors and specimens is limited, as is the number of specimens that can be tested biomechanically. Of course, larger numbers would be desirable here. Other biomechanical studies often use similar numbers of pairs. Between 8 and 12 pairs are regular sample sizes^34–36^. The tests performed here were standardized both with the HT and with the STT and were consistently performed with the same forces. For HT, we know that it is used intraoperatively by surgeons with very different forces. However, to be able to compare the test methods, this standardization is necessary. The diastasis was monitored with a 3D camera that can detect movements with micrometer accuracy. This is not possible under fluoroscopic viewing of the ankle mortise at all. Therefore, the critical integration of the results for clinical practice must be done. Under X-ray control, even a few degrees of deviation from the ideal setting of the mortise view can bias the results of the radiological parameters (tibiofibular overlap/clear space, medial clear space).

Minor injuries with one or two ruptured ligaments are difficult to detect with the devices used in our study. The question of whether these one or two ligament injuries are stability-relevant and require surgical stabilization has not been answered until now. In a cadaver study, it was shown that direct visualization and measurement of diastasis in the syndesmosis appear to be more sensitive than radiographic imaging^37^. A combination of standardized applied force and direct visualization of the anterior syndesmosis with, if necessary, measurement of the syndesmosis at the ventral entrance of the incisura could be a good diagnostic tool in cases of unclear findings in pre- or intra-operative imaging. In the future, further studies will be necessary to promote the possibility of clinical use of STT. The first step should be to evaluate the influence of the direction of stress on the diastasis in the ankle joint. Furthermore, the STT must be adapted to the optimal force application for detecting instabilities of the syndesmosis and a standardized method must be found to identify relevant instability. It may also be possible to measure diastases directly on the instrument regardless of the correct setting of the mortise view. Furthermore, clinical studies investigating for example the association between the extent of syndesmosis damage to functional outcomes would benefit from the use of the STT.

In conclusion, reliable visual detection of an incomplete syndesmosis injury is hardly possible, even with higher degrees of injury and even with a standardized procedure. A Complete injury to the syndesmosis complex can be detected more sufficient with the STT than with the HT. The STT shows significantly better differentiability in syndesmosis injuries compared to the native condition. A macroscopically visible diastasis of 2 mm is not achieved in every case, even with higher-grade ligamentous injuries to the syndesmosis. Overall, the results show that the decision to stabilize the syndesmosis should not be based only on intraoperative HT. Definitive evidence of instability seems often only possible in cases of maximum instability. The direction of the force application, which was technically different between both tools, seems to matter on the diastasis, which should not be underestimated and should be investigated in more detail in the future.

## Data Availability

The datasets generated during and analyzed during the current study are available from the corresponding author upon reasonable request.
